# Proliferation, apoptosis and their regulatory protein expression in colorectal adenomas and serrated lesions

**DOI:** 10.1371/journal.pone.0258878

**Published:** 2021-11-11

**Authors:** Jane C. Figueiredo, Michael N. Passarelli, Wei Wei, Dennis J. Ahnen, Jeffrey S. Morris, Lynda Corley, Trupti Mehta, Angela N. Bartley, Gail McKeown-Eyssen, Robert S. Bresalier, Elizabeth L. Barry, Ajay Goel, Goretti Hernandez Mesa, Stanley R. Hamilton, John A. Baron

**Affiliations:** 1 Department of Medicine, Samuel Oschin Comprehensive Cancer Institute, Cedars-Sinai Medical Center, Los Angeles, California, United States of America; 2 Department of Epidemiology, Geisel School of Medicine at Dartmouth, Lebanon, New Hampshire, United States of America; 3 Taussig Cancer Institute, The Cleveland Clinic, Cleveland, Ohio, United States of America; 4 Division of Gastroenterology and Hepatology, University of Colorado School of Medicine, Denver, Colorado, United States of America; 5 Department of Biostatistics, Epidemiology & Informatics, University of Pennsylvania, Perelman School of Medicine, Philadelphia, Pennsylvania, United States of America; 6 Division of Pathology and Laboratory Medicine, Department of Pathology, The University of Texas MD Anderson Cancer Center, Houston, Texas, United States of America; 7 St. Joseph Mercy Hospital, Ann Arbor, Michigan, United States of America; 8 Dalla Lana School of Public Health, University of Toronto, Toronto, Canada; 9 Department of Gastroenterology, Hepatology, and Nutrition, University of Texas M.D. Anderson Cancer Center, Houston, Texas, United States of America; 10 Center for Gastrointestinal Research, Center for Translational Genomics and Oncology, Baylor Scott & White Research Institute and Charles A. Sammons Cancer Center, Baylor Research Institute and Sammons Cancer, Dallas, Texas, United States of America; 11 Department of Pathology, City of Hope National Cancer Center, Duarte, California, United States; 12 Department of Gastroenterology, University Hospital of the Canary Islands, La Laguna, Tenerife, Spain; 13 Department of Medicine, University of North Carolina at Chapel Hill, Chapel Hill, North Carolina, United States of America; Universitat des Saarlandes, GERMANY

## Abstract

**Background:**

Adenomas and serrated lesions represent heterogeneous sets of early precursors in the colorectum with varying malignant potential. They are often distinguished by their histopathologic differences, but little is known about potential differences in regulation of epithelial proliferation and apoptosis.

**Methods:**

We conducted a protein expression analysis using tissue microarrays of 625 colorectal adenomas and 142 serrated lesions to determine potential differences in regulation of epithelial proliferation and apoptosis. We quantitated proliferation with Ki-67; apoptosis with activated caspase-3 (CASP3); up- and down-regulators of proliferation with cyclin D1, p16^INK2^, and p21^Cip1^; and apoptosis regulators with BAX, BCL2, and survivin. Linear mixed effects models and circos diagrams were used to determine relationships among expression and lesion characteristics.

**Results:**

Adenomas had a significantly higher CASP-3 labeling index (LI) than serrated lesions, resulting in a lower net growth ratio (Ki-67 LI/activated CASP-3 LI, p-value<0.0001). Cyclin D1 LI, p16 LI and p21 LI were lower in adenomas compared to serrated lesions, while expression of both BCL2 and BAX were higher (p <0.001). Among adenomas, cyclin D1 LI and p16 LI levels increased with greater villous component, and the highest BAX expression was detected in adenomas larger than 2 cm (both p<0.0001). Right-sided adenomas had higher CASP3 LI than left colorectal adenomas (p = 0.008). Significant differences in cyclin D1 LI, p21 LI and survivin LI were also observed across histopathologic subtypes of serrated lesions.

**Conclusions:**

Our findings demonstrate different patterns of regulatory protein expression in adenomas than serrated lesions, especially involving apoptosis.

**ClinicalTrials.gov Identifier:** NCT00272324

## Introduction

Colorectal adenocarcinoma, the second most common cause of cancer deaths [1], is clearly a heterogeneous disease. A useful, although incomplete, categorization is to distinguish cancers arising from the adenomatous and serrated pathways. Adenomas are precursors to most microsatellite-stable cancers, progressing through chromosomal instability to invasive cancer [2]. These precursor lesions are typically sub-classified on the basis of their size and histopathologic appearance. Larger adenomas and those with high-grade dysplasia and villous or tubulovillous architecture are well known to have a higher risk of progression to carcinoma than smaller adenomas or those with tubular histology and low-grade dysplasia [3].

Serrated lesions are a heterogeneous set of early precursor lesions, comprising hyperplastic polyps (HPPs); sessile serrated lesions (SSLs), variously termed sessile serrated adenoma, sessile serrated polyp, and sessile serrated adenoma/polyp without or with dysplasia; traditional serrated adenoma (TSA); and serrated lesions, unclassified [4]. SSLs are the precursors to most colorectal cancers exhibiting high levels of microsatellite instability (MSI-H)/deficient mismatch repair (dMMR) and the CpG island methylator phenotype CIMP) [5–8]. The molecular features of TSAs are not as clearly defined as SSLs, but include KRAS or BRAF mutations along with variable levels of CpG island hypermethylation (CPG methylator phenotype, CIMP) [9]. Unlike some other SSL, these tumors do not appear to progress through MLH1 mismatch repair gene hypermethylation and MSI [9]. Unlike SSLs and TSAs, serrated lesions classified as HPPs are thought to have limited immediate potential to progress to carcinoma.

A few relatively small studies have investigated the cellular kinetics of adenomas and serrated lesions by comparing expression patterns of proteins involved in cellular proliferation and apoptosis. Several of these studies show lower rates of apoptosis in serrated lesions compared to adenomas [10–15], and it has been suggested that this lower apoptotic rate leads to crypt cell accumulation causing the characteristic saw-toothed structure of serrated polyps [16, 17]. Lower proliferative activity in serrated lesions compared to adenomas has also been reported [10, 11, 14, 18–20] and, importantly, the ratio of proliferation to apoptosis that is described as the net growth ratio may be higher in serrated lesions compared to adenomas [14, 15]. All of these studies have been based on small convenience collections and did not systematically examine regulatory molecules for proliferation and apoptosis in histopathologic categories or location in the colorectum. We therefore investigated these protein expression characteristics in a large set of colorectal adenomas and serrated lesions removed from participants in a randomized chemoprevention clinical trial [21, 22].

## Materials and methods

### Study population

Routine specimens obtained from patients undergoing screening or surveillance colonoscopy were used for our study. These were baseline polyps for participants enrolled in the Aspirin/Folate Polyp Prevention Study, a randomized adenoma chemoprevention trial [21, 22]. Potential participants were recruited between July 1994 and March 1998 at nine clinical centers in the United States and Canada. Eligible individuals aged 21–80 years had one or more recent adenomas, and no known polyps remaining in the colorectum after complete colonoscopy. The Committee for the Protection of Human Subjects at Darmouth approved the study protocol and all participants signed informed consent.

### Tissue microarray (TMA) construction

Tissue blocks were requested for all baseline adenomas and right-sided serrated lesions, and a 50% random sample of the numerous baseline left-sided serrated lesions, most classified as HPPs. Tissue blocks for all but two study centers were sent to The University of Texas M.D. Anderson Cancer Center (UT-MDACC) for TMA construction. The University of Toronto and the University of Colorado prepared TMA blocks for their samples and provided the completed recipient blocks. A total of 18 TMA blocks was constructed: 15 at UT-MDACC, two at the University of Toronto, and one at the University of Colorado.

TMAs were constructed at each of the three laboratories with a manual Beecher coring instrument (Sunprarie, WI). When dysplasia was present, the most advanced area was selected first for sampling. To accommodate differences in lesion size and intra-tumor heterogeneity, about 5% of the area of each lesion was sampled. In blocks requiring multiple cores to ensure adequate sampling, the cored sites were equally spaced around the tissue after the first sample from the area of most advanced dysplasia. The cores were obtained serially and inserted into a recipient TMA block without access to any clinical characteristics of the patient. Four control cores (two each from a single colorectal adenocarcinoma and a spleen) were included in each TMA recipient block prepared at the UT-MDACC to permit assessment of the consistency of immunohistochemistry among the blocks and provide orientation of the slides for computer-assisted image analysis.

We obtained participant consent for a collection of 2,548 baseline lesion specimen blocks that met our selection criteria. Of those, we were able to retrieve 1,595 (63%); 306 (12.0%) were not released by the pathology laboratories; 225 (8.8%) had been destroyed or lost; 12 (0.5%) had insufficient tissue remaining in the lesion block; and 410 (16.1%) were not obtained due to study coordinator error or unknown reasons. The 1,595 blocks contained 1,861 lesions, but 731 of those lesions had insufficient remaining tissue for coring. A final total of 2,646 cores from the available 1,130 lesions were acquired and placed into the 18 TMA blocks.

Once the TMAs were prepared, each core was reviewed on an H&E-stained slide of each completed TMA block by two gastrointestinal pathologists (ANB and SRH). The histopathologic classification for adenomas and for serrated lesions from the World Health Organization 2019 edition [4] were applied. Each adenoma was classified as tubular, tubulovillous, or villous, and each serrated lesion as HPP (without subcategorization), SSL (formerly sessile serrated adenoma/polyp), SSLD (sessile serrated lesion with dysplasia, formerly sessile serrated adenoma/polyp with cytologic dysplasia), TSA (traditional serrated adenoma), or serrated lesion of uncertain type [4, 8]. The TMA core classification was compared to that obtained from review of the corresponding lesion scout slide when available. Any discrepancies were either reconciled or flagged for exclusion from statistical analyses because the core was deemed of questionable quality (e.g. cautery artifact) or had missed its sample target within the lesion. Our final analysis utilized data derived from 1,972 cores from 767 baseline lesions (625 adenomas and 142 serrated lesions) from 486 participants.

### Immunohistochemistry for proliferation, apoptosis, and regulatory proteins

We examined the proteins that measure or regulate cell proliferation (Ki-67, cyclin D1, p16, p21) and apoptosis (CASP3, BAX, BCL2, survivin) as listed in **S1 Table**. Immunohistochemistry was performed with validated antibodies in the UT-MDACC CLIA-accredited clinical Immunohistochemistry Laboratory, Deparaffinized 4-micrometer sections from the TMA blocks were stained using standardized avidin-biotin-peroxidase complex methods on automated instruments (Leica Bond or Ventana) after elimination of endogenous peroxidase and using antigen retrieval, as previously described [23]. Methodological details are summarized in **S2 Table**. The histologic sections for each antibody from all TMAs were stained in one batch to eliminate batch effects. 3,3’-diaminobenzidine was the chromogen, and light Mayer’s hemotoxylin was used as the counterstain. We included known positive and negative tissues as controls for all antibodies used in each immunohistochemistry run. Each slide was evaluated by a gastrointestinal pathologist (ANB or SRH) for immunohistochemistry quality.

### Image analysis

Computer-assisted digital image analysis of lesional epithelium in each TMA immunohistochemistry slide was performed for quantitation of marker protein expression with the Aperio system (Morrisville, NC). All immunohistochemistry slides were scanned at 20X using the Aperio AT2 scanner and ScanScope software. Individual maps were used for identification of each unique core for all stained slides. Quantitation of biomarkers was done with the AT2 instrument and ImageScope software. Aperio Genie Pattern Recognition software was used to identify lesional epithelium for evaluation, and the Aperio Genie nuclear v.9.1 and Color Deconvolution v9 algorithms were used to create a custom expression classifier for nuclear positivity. The Genie Pattern Recognition software was then used to extract digitally the multiple sampled areas representing various lesions to create a montage consisting of all selected acquired areas. The same steps were then repeated to identify stroma and other unwanted tissue areas to isolate the lesion individually for quantitation and at the same time represent the entire area of each core for quantitation. Once all of the samples for quantitation were selected, the montage was saved in a custom nuclear Genie Training Macro that was tested to assess the accuracy of separating lesional epithelium from unwanted areas. The performance was deemed satisfactory when >90% mean sensitivity and specificity were obtained for the lesion and unwanted areas. The process was then repeated for all cytoplasmic biomarkers.

### Statistical analysis

For the nuclear proteins, the primary measure was the percentage of positive nuclei among all lesional epithelial nuclei (labeling index, LI). For the cytoplasmic proteins, the percentage of total lesional epithelial area measured in pixels that had positive staining was the primary data element. Immunostaining for Ki-67 was used as an index of proliferation, and cleaved caspase-3 (CASP3) as an index of apoptosis [24, 25]. To stabilize the variance of the observations and make model residuals more independent and normally distributed, analyses were performed after log transformations (p16, p21, CASP3, BCL2) or square root transformations (Ki-67, cyclin D, BAX), based on raw data distributions. Values were transformed back to the original scale for presentation. No transformation was required for survivin expression data. The BCL2/BAX ratio was computed as a measure of anti- versus pro-apoptotic control, and the Ki-67/CASP3 ratio (termed “net growth ratio”) as a measure of proliferation versus apoptosis.

We explored whether the protein expression in the baseline lesions of patients in the trial varied by lesion characteristics [histopathology, size as estimated by the endoscopist, and location defined as left-sided (distal) for splenic flexure to the rectum or right-sided (proximal) for ceum through transverse colon]. To account for the nested relationships among cores, lesions and participants, and the variability in original tissue block preparations and TMA blocks, we fit linear mixed effects models with participant, cores, TMA block, and source pathology laboratory as random-effect factors, and age categories, sex, lesion type and lesion characteristics as fixed effects. Fixed effects were estimated using model-based least squared means from the mixed effects models, with degrees of freedom for the various factors estimated using a Satterthwaite approximation.

We performed separate evaluations for each protein examined. Sample sizes in data tables varied due to random missing or poorly oriented TMA cores. Since we focused on eight specific proteins that have strong *a priori* rationale for their importance in carcinogenesis of the colorectum, we report p-values in the tables unadjusted for multiple statistical comparisons. P-values for a linear trend were calculated by modelling adenoma size as a continuous variable for tubular adenomas and adenoma histology as a grouped continuous variable with a common slope between groups (tubular, tubulovillous, villous histology). In analyses by histology, two subgroup tests were performed, comparing TSAs with other serrated lesions and SSLs with HPPs. All tests were two-sided with p<0.05 considered statistically significant. Statistical analysis was carried out using SAS version 9.3 (SAS Institute, Cary, NC). P<0.05 was considered statistically significant.

**Fig 1:**
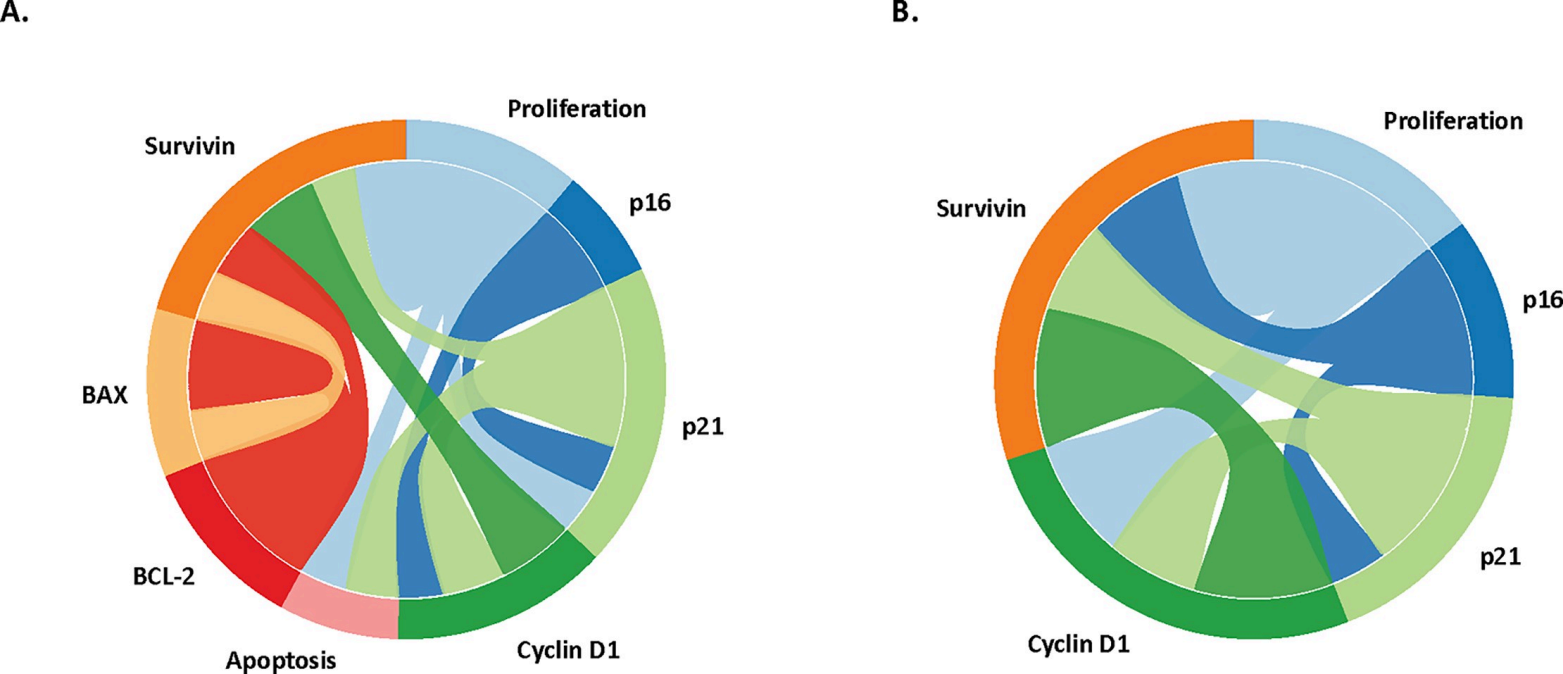
Circos diagrams visualizing the correlations of expression of proteins in tubular adenomas (**Panel A**) and hyperplastic polyps (**Panel B**). Correlated expression of BAX and BCL2 and evidence of apoptosis are present in adenomas, but not in hyperplastic polyps.

Circos diagrams were prepared to visualize the correlations of expression of proteins separately in tubular adenomas and HPPs (**Fig 1**). The correlation coefficient was calculated for each pair of proteins for 546 tubular adenomas and 101 HPPs (**S3 Table**). A presentation threshold of r> 0.24 was used to simplify the Circos plots and focus on the highest magnitude correlations. P-values for contrast were calculated. The correlation coefficients were entered into software E90E50fx from www.circos.ca to construct the diagrams.

## Results

Among participants, 455 had at least one core from a baseline adenoma, including 269 with two or more cores; 102 individuals had a least one core from a baseline serrated lesion, including 46 with two or more cores (**Table 1**). A total of 71 individuals contributed cores from both adenomas and serrated lesions at baseline.

**Table 1:** Demographic information on the study participants.

Characteristic		N	%
Age	< 50	116	24
	51–60	171	35
	61–65	86	18
	66+	113	23
Sex	Female	179	37
	Male	307	63
Body Mass Index (kg/m^2^)	<25	140	29
	25–29	229	47
	> = 30	116	24
	Missing	1	-
Smoking Status	Never	205	42
	Former	206	43
	Current	73	16
	Missing	2	-
Alcohol Use	None	145	31
	<2 drinks/day	286	61
	2+ drinks/day	37	8
	Missing	18	-

### Proliferation and apoptosis in adenomas and serrated lesions

The Ki-67 LI was higher in serrated lesions compared to adenomas (p = 0.04), while that for CASP-3 was lower (p = 0.0004, **Table 2**). Consequently, the net growth ratio was significantly lower in adenomas compared to serrated lesions (p<0.0001). Adenomas were found to have lower LIs of the pro-proliferative cyclin D1 protein and anti-proliferative proteins p16 and p21 as contrasted with serrated lesions. Adenomas also displayed higher pro-apoptotic BAX and anti-apoptotic BCL2 expression compared to serrated lesions.

**Table 2:** Mean protein expression levels comparing adenomas to serrated lesions.

	Precursor Histology		
	**Adenomas**	Serrated Lesions	P ^for difference^
**No. subjects**	455	102	
**No. polyps**	625	142	
**No. cores**	1770	202	
**Proliferation (LI)**			
Ki-67	18.12 (13.67–23.19)	20.65 (15.47–26.52)	0.04
Cyclin D1	26.53 (22.04–31.44)	38.98 (32.75–45.75)	<0.0001
p16	20.74 (15.66–26.53)	29.82 (22.77–37.83)	<0.0001
p21	11.87 (9.19–14.90)	23.67 (19.26–28.54)	<0.0001
**Apoptosis (LI)**			
CASP3	1.62 (1.23–2.07)	1.15 (0.77–1.60)	0.0004
BAX	25.22 (19.98–31.08)	19.54 (14.24–25.66)	0.001
BCL2	15.99 (10.09–23.24)	9.74 (5.04–15.98)	<0.0001
Survivin	66.33 (60.75–72.13)	68.21 (61.76–74.98)	0.30
**Ratio**			
Ki-67/ CASP3	15.77 (11.15–21.19)	37.34 (28.03–47.98)	<0.0001
BCL2/BAX	0.87 (0.56–1.25)	1.02 (0.62–1.52)	0.24

LI, labeling index; Adjusted Means (95% confidence intervals) for protein expression in baseline polyps; adjusted for size, age, and gender as fixed effects in all models, and adjusted for pathology lab, subject, cores, and TMA block as random effects.

### Pathologic characteristics and location of adenomas

Although the LI for Ki-67 was similar across the spectrum of villous architecture, cyclin D1 and p16 LIs increased with greater villous component (p<0.001, **Table 3**). There was no corresponding significant difference in the net growth ratio, but the BCL2/BAX ratio decreased significantly with villous histology (p = 0.001). In terms of anatomic location, adenomas located in the proximal colon had notably higher CASP3 LI compared to those in the distal colorectum (p = 0.0005), but similar levels of apoptosis-regulating proteins and similar proliferation. The net growth ratio was consequently higher in distal lesions (p = 0.008).

**Table 3:** Mean protein expression levels by histopathologic subtype and location among all adenomas.

	Histology				Location		
	**Tubular**	**Tubulovillous**	**Villous**	**P ^for trend^**	**Proximal**	**Distal**	**P ^for difference^**
**No. subjects**	415	49	5		182	241	
**No. polyps**	546	67	12		221	304	
**No. cores**	1259	437	74		458	1021	
**Proliferation (LI)**							
Ki-67	18.34 (13.72–23.63)	17.27 (12.55–22.75)	21.97 (14.10–31.56)	0.92	19.41 (14.56–24.94)	18.06 (13.48–23.31)	0.16
Cyclin D1	25.30 (20.56–30.53)	28.25 (22.81–34.27)	42.10 (31.15–54.70)	0.001	26.00 (21.52–30.90)	26.68 (22.29–31.46)	0.58
p16	19.08 (14.15–24.75)	24.49 (18.33–31.54)	41.74 (27.42–59.06)	<0.0001	22.12 (16.33–28.78)	21.98 (16.38–28.41)	0.93
p21	11.72 (9.03–14.76)	12.40 (9.37–15.86)	12.37 (7.34–18.70)	0.50	12.45 (9.68–15.57)	11.15 (8.63–13.99)	0.08
**Apoptosis (LI)**							
CASP3	1.57 (1.19–1.99)	1.76 (1.32–2.27)	1.19 (0.60–1.99)	0.71	1.81 (1.42–2.23)	1.41 (1.09–1.77)	0.0005
BAX	23.70 (18.38–29.69)	30.31 (23.57–37.90)	29.30 (17.93–43.46)	0.005	26.12 (20.31–32.66)	24.76 (19.32–30.88)	0.39
BCL2	16.24 (10.06–23.89)	17.09 (10.49–25.29)	6.72 (1.78–14.84)	0.27	16.23 (10.24–23.60)	16.54 (10.57–23.84)	0.79
Survivin	65.92 (60.29–71.81)	67.78 (61.59–74.27)	69.17 (58.41–80.84)	0.26	65.57 (59.37–72.07)	66.23 (60.15–72.61)	0.62
**Ratio**							
Ki-67/ CASP3	16.04 (11.67–21.10)	16.28 (11.16–22.38)	24.54 (12.89–39.92)	0.38	13.67 (9.43–18.69)	17.83 (13.20–23.16)	0.008
BCL2/BAX	1.04 (0.70–1.43)	0.68 (0.39–1.06)	0.18 (0.00–0.70)	0.0003	0.92 (0.58–1.34)	0.99 (0.64–1.41)	0.52

LI, labeling index; Adjusted Means (95% confidence intervals) for protein expression in baseline adenomas; Results obtained from a mixed model accounting for correlation between cores within polyps, polyps within subjects, pathology laboratory (random effect) and TMA block (random effect) and adjusting for polyp size (<1 cm, 1–1.9 cm, 2+ cm). P for trend for adenoma histology is from a mixed model that includes adenoma histology coded as a grouped continuous variable with a common slope between groups.

Among tubular adenomas, none of the apoptosis regulators except BAX was associated with adenoma size (p-trend<0.0001, **Table 4**). The positive association with BAX led to an inverse association for the BCL2/BAX ratio with increasing adenoma size (p-trend<0.0001).

**Table 4:** Mean protein expression levels by size of tubular adenomas.

	Tubular Adenoma Size			
	**< 1 cm**	**1–1.9 cm**	**> = 2 cm**	**P ^for trend^**
**No. subjects**	274	94	16	
**No. polyps**	343	102	22	
**No. cores**	481	457	197	
**Proliferation (LI)**				
Ki-67	18.46 (13.67–24.00)	17.59 (12.79–23.06)	20.65 (14.62–27.71)	0.86
Cyclin D1	25.32 (20.68–30.42)	25.97 (21.11–31.35)	27.73 (21.34–34.95)	0.35
p16	18.30 (13.18–24.27)	17.32 (12.21–23.32)	18.08 (11.75–25.76)	0.76
p21	11.68 (8.88–14.85)	11.05 (8.26–14.25)	14.15 (10.23–18.70)	0.92
**Apoptosis (LI)**				
CASP3	1.50 (1.14–1.90)	1.44 (1.07–1.86)	1.63 (1.11–2.25)	0.90
BAX	16.93 (12.36–22.22)	24.71 (18.89–31.31)	33.50 (24.77–43.54)	<0.0001
BCL2	17.34 (10.88–25.30)	18.51 (11.72–26.85)	14.10 (7.65–22.49)	0.79
Survivin	66.55 (60.47–72.91)	66.51 (60.28–73.05)	66.43 (58.87–74.45)	0.48
**Ratio**				
Ki-67/ CASP3	17.78 (13.07–23.21)	16.20 (11.47–21.75)	15.99 (9.60–23.99)	0.51
BCL2/BAX	1.45 (1.03–1.94)	0.98 (0.63–1.41)	0.64 (0.29–1.13)	<0.0001

LI, labeling index; Adjusted Means (95% confidence intervals) for protein expression in baseline adenomas; Results obtained from a mixed model accounting for correlation between cores within polyps, polyps within subjects, pathology laboratory (random effect) and TMA block (random effect) and adjusting for histology (tubular, tubulovillous, villous). P for trend is from a mixed model that includes tubular adenoma size as a continuous variable.

### Pathologic characteristics of serrated lesions

There were no differences in Ki-67 or CASP3 LI’s between SSLs and HPPs, and the proliferation and apoptosis regulatory proteins did not differ either (**Table 5**). Ki-67 and the proliferation-regulating proteins were also similar in SSLs and SSLDs, but the latter had a significantly higher CASP3 LI, though with no notable differences in apoptosis-regulating proteins, TSAs had lower levels of all of the analytes measured. This was particularly marked for the CASP3 LI, though only the differences for cyclin D1 and survivin were statistically significant.

**Table 5:** Mean protein expression levels by histopathologic subtype of serrated lesions.

	**Histology**				**p for HPP**	**p for SSL**	**p for TSA**
	**Hyperplastic polyp**	**Sessile serrated lesion**	**Sessile serrated lesion with dysplasia**	**Traditional Serrated Adenoma**			
**No. subjects**	78	15	20	2			
**No. polyps**	101	15	23	2			
**No. cores**	113	45	40	3	**v. SSL**	**v. SSLD**	**v. other**
**Proliferation (LI)**							
Ki-67	22.28 (14.44–31.81)	20.14 (11.73–30.82)	20.21 (13.14–28.80)	15.74 (3.61–36.42)	0.53	0.93	0.50
Cyclin D1	47.32 (37.89–57.80)	39.84 (29.82–51.32)	34.08 (26.92–42.09)	18.79 (6.27–38.02)	0.10	0.33	0.02
p16	29.59 (16.84–45.91)	29.58 (15.74–47.75)	32.83 (19.60–49.46)	20.84 (3.18–53.97)	0.98	0.58	0.42
p21	28.40 (20.97–36.96)	29.70 (21.29–39.52)	18.06 (12.50–24.64)	15.29 (5.37–30.29)	0.70	0.003	0.25
**Apoptosis (LI)**							
CASP3	1.30 (0.51–2.44)	1.08 (0.31–2.31)	2.14 (1.12–3.49)	0.37 (0.07–2.16)	0.50	0.04	0.12
BAX	15.63 (8.34–25.20)	20.19 (10.74–32.61)	25.26 (16.26–36.24)	6.20 (0.02–26.17)	0.23	0.33	0.14
BCL2	10.86 (5.47–18.09)	12.05 (5.70–20.75)	17.60 (10.86–25.97)	5.31 (0.03–19.64)	0.67	0.13	0.18
Survivin	71.69 (59.74–84.73)	67.10 (54.36–81.19)	70.36 (58.88–82.87)	32.53 (16.41–54.10)	0.55	0.55	0.002
**Ratio**							
Ki-67/ CASP3	47.66 (15.63–97.09)	33.11 (5.60–83.57)	13.91 (0.94–42.15)	51.80 (0.06–200.4)	0.33	0.21	0.62
BCL2/BAX	1.71 (0.48–3.71)	1.55 (0.36–3.56)	1.23 (0.25–2.95)	1.76 (0.11–5.41)	0.69	0.48	0.79

HP, hyperplastic polyp; LI, labeling index; SSL, sessile serrated lesion; TSA, traditional serrated adenoma; Adjusted Means (95% confidence intervals) for protein expression in baseline serrated lesions; Results obtained from a mixed model accounting for correlation between cores within polyps, polyps within subjects, pathology laboratory (random effect) and TMA block (random effect) and adjusting for polyp size (<1 cm, 1–1.9 cm, 2+ cm).

There were no notable differences in LIs for Ki-67 or CASP3 according to location among serrated lesions.

### Correlation of proliferation, apoptosis, and regulatory protein expression

As might be expected, the Ki-67 LI was positively correlated with Cyclin D1 in both tubular adenomas and hyperplastic polyps, especially the latter, where the correlation was 0.50 (p<0.0001) (**S3 Table**, **Fig 1)**. The Ki-67 LI also tended to be positively correlated with those for its negative regulators p16 and p21 in both types of polyps. The CASP3 LI was essentially uncorrelated with any of its regulators in both types of polyps, but was positively correlated with p16 and p21 in tubular adenomas only. In tubular adenomas, but not in HPPs, pro-apoptotic BAX was strongly correlated with anti-apoptotic BCL2 (r = 0.48, p<0.0001), The survivin LI was highly correlated in both types of lesions with the KI-67 LI and with each of the proliferation-related analytes except for p16 in tubular adenomas. In contrast, it was essentially uncorrelated with CASP3 in both types of lesions and uncorrelated with BAX and BCl2 in HPPs.

## Discussion

We confirm in a large series of carefully annotated specimens that serrated lesions have substantially lower apoptotic rates and higher proliferation/apoptosis ratios compared to adenomas, changes that would favor cellular accumulation in serrated lesions. We also found that the expression patterns of regulatory proteins in adenomas are substantially more complex than in serrated lesions. SSLs have similar expression patterns as HPPs, but the dysplastic areas of SSLs with dysplasia have apoptotic rates higher than other serrated lesions and similar to those found in conventional adenomas. The absence of inverse relationships among proliferation, apoptosis, and expression of regulatory proteins indicates that activation of counterbalancing mechanisms, rather than negative feedback to suppress pathways, is important in these precursors to colorectal adenocarcinoma.

Previous studies comparing serrated lesions and adenomatous polyps have been small series published before the current histopathologic classification of subtypes of serrated lesions. In these older studies, “serrated adenomas” very likely refer to lesions that would currently be called SSLs [8]. In aggregate, the reported studies suggest that these lesions have higher levels of proliferation than hyperplastic polyps and probably lower levels than adenomas [10, 11, 14, 15, 18, 19, 26, 27]. These studies also found that serrated lesions had lower apoptotic indices than adenomas, and that hyperplastic polyps had lower indices than “serrated adenomas” [10–15, 18, 28]. The lower apoptotic measures and the higher Ki-67/CASP3 ratio in serrated lesions in our study support the longstanding concepts that the net growth ratio in serrated polyps is substantially lower than in adenomas, and that SSLs have a preferential failure of physiologic cell death rather than a substantial excess of cell production [11]. The limited surface area in the crypts and glands along with failure of cell death may be the functional basis for the serrations that are characteristic of this pathway.

Few previous studies have examined the expression levels of specific cell cycle regulatory proteins in precursor lesions. A small study [29] showed higher p16 immunohistochemistry staining in adenomas than in HPPs, in contrast to our much larger study which observed higher p16 levels in serrated lesions compared to adenomas. In our study, p16 levels also increased with villous histology. Regarding p21 expression, consistent with our data, Mitomi et al. [12] observed higher p21 expression in serrated lesions than in adenomas. Similar findings were also reported by Jiao et al. [30]. Although p21 is known to inhibit cell cycling, it can also inhibit apoptosis, perhaps explaining the reduced apoptosis in serrated lesions [31]. Also, in concordance with previous studies [11, 13, 19, 32], we observed lower BCL2 expression in serrated lesions than in adenomas. Lower levels of BAX have also been reported in HPPs compared to other types of serrated lesions as well as adenomas [11, 13, 14]. Lower levels of both pro- and anti-apoptotic proteins in serrated lesions do not explain the markedly lower apoptotic rate in these lesions, suggesting that other regulatory factors must also be involved in apoptosis induction.

Among adenomas, those with villous histology had a proliferation LI similar to tubular and tubulovillous adenomas, but had higher cyclin D1 levels. Other studies have shown that Ki-67 and cyclin D1 levels are higher in carcinomas compared to adenomas [28, 33–35]. Tubulovillous adenomas also showed a strikingly low expression of pro-apoptotic BCL2 and a low pro- vs anti-apoptotic ratio, BCL2/BAX, despite small differences in the CASP3 LI. Large tubular adenomas (two cm or greater) also exhibited the highest level of BAX and consequently the lowest BCL2/BAX ratio. Other studies have observed lower BCL2 expression in villous compared to tubular adenomas, as well as in carcinomas compared to adenomas [28, 34]. These results suggest that BCL2 expression decreases in the course of the adenoma-to-carcinoma transition, and that susceptibility to apoptosis also diminishes. Furthermore, the low BCL2/BAX ratio, which should reflect relative promotion of apoptosis as opposed to the lower measured apoptotic rates in villous lesions, implies other regulatory elements are also affecting apoptosis in the lesions.

Among serrated lesions, differences in proliferation, apoptosis and regulatory protein levels occurred in different types of lesions. We found SSLs to be similar to HPPs with regard to proliferation, apoptosis, and their regulatory proteins, but that SSLs with dysplasia differed from SSLs without dysplasia in having lower p21 and higher CASP3. The proliferation/apoptosis ratio of SSLs with dysplasia was numerically lower than that for SSLs (though not significantly so), and similar to that of adenomas, suggesting similar net cell kinetics.

The small number of TSL lesions we included were distinct from other serrated lesions in having lower levels of each of the analytes studied. Although in relative terms this was most marked for CASP3 and BAX, the differences were statistically significant only for cyclin D, and survivin. There is little prior data regarding TSLs. Bettington and colleagues also found a low Ki-67 index in TSLs, but nuclear or cytoplasmic p16 staining was no different than in tubulovillous adenomas [20].

Our finding that the CASP3 LI is higher in adenomas located in the right colon than those in the left colorectum is a novel observation. There are other well-documented histopathologic and molecular differences in the characteristics of tumors located on the right and left side of the colorectum, including, for example, the prevalence of *BRAF* and *KRAS* mutations and CIMP hypermethylation [36, 37]. Similarly, precursor lesions also exhibit a preferential location in the colorectum: SSLs occur more frequently in the right colon, while TSAs and HPPs are most often found in the left colon and rectum [8]. The biological basis for these right/left differences is not understood fully, but one fundamental difference is the luminal environment between the right and left colon. Differences in embryology could also be responsible for a field effect that mediates these differences. Only one previous study that examined apoptotic index and anatomical location found no relationship [38]. Further research is therefore needed.

Our study has notable strengths. We evaluated a large number of precursor lesions in well-characterized clinical trial participants. As part of a clinical trial, the colonoscopy and pathology findings were collected in a standardized manner. Our series of precursors was large enough to assess proliferation and apoptosis as a function of histopathologic subtypes and of size and anatomic location within the colorectum. We had detailed data collection protocols, and all the precursor lesions obtained for the TMAs were re-reviewed centrally and classified by the 2019 WHO criteria for adenomatous and serrated lesions [4]. We used standardized immunohistochemical methods, well-established robust antibodies, and a quantitative computer-assisted image analysis system.

Our study also has limitations. This clinical trial admitted only patients with a personal history of adenomas, as assessed by local pathologists, so that our findings may not apply to serrated lesions that occur in the absence of a synchronous adenoma. A percentage of the lesions available were too small to sample, resulting in exclusion of these small precursors; therefore, our results may be generalizable only to larger lesions. The specimens that we analyzed were obtained from many different pathology laboratories. Thus, we could not optimize our antibodies and analytic procedures for varying pre-analytic tissue fixation and histologic processing. Although this variability likely added variability to our measurements, systematic errors are unlikely. We had only 2 TSA lesions in our analysis, with 3 cores, resulting in wide confidence limits for all the measurements for this type of lesion.

An important limitation of TMA methodology is that it uses only a small fraction of a tissue specimen, which may not be representative, especially for antigens with heterogeneous expression patterns in tumors. Also, we were unable to examine any of the biomarkers in spatially distributed regions of the glands. However, a validation study in colorectal adenomas [33] has evaluated the validity of a TMA constructed from colorectal adenoma tissue for selected immunohistochemical expression including Ki-67 and cyclin D1. Results from that study showed that colorectal adenomas exhibited zonal, heterogeneous expression patterns for all five markers. The concordance rates for the semi-quantitatively evaluated biomarkers in TMAs and whole sections were quite good, ranging from 87% to 93%, with corresponding kappa statistics of 0.77 to 0.90 [33]. We used standard quality control procedures to ensure accuracy of the immunostaining in our study.

## Conclusions

Our findings emphasize the complexity and heterogeneity between adenomas and serrated lesions. From a clinical perspective, understanding underlying mechanistic differences between precursors to adenocarcinoma in the colon, may provide greater insight into which lesions should be followed clinically. This goal is underscored by the recently updated U.S. Multi-Society Task Force guidelines for follow up after colonoscopy and polypectomy [39]. For example, these guidelines suggest that individuals with 1 to 2 SSPs (SSLs) <10 mm have surveillance at a slightly shorter interval (5–10 years) compared to those with 1–2 tubular adenomas <10 mm (7–10 years), while those with an SSP with dysplasia undergo surveillance at 3 years. These recommendations are based on perceived risk, but the quality of evidence and strength of recommendation for serrated lesions are considered very low and weak, respectively [40]. A better understanding of the epithelial kinetics of these lesions may help inform such surveillance practices.

## Supporting information

S1 Table List of proliferation and apoptosis proteins examined in this study. (DOCX)

S2 Table List of immunohistochemistry reagents and methods. (DOCX)

S3 Table Correlation coefficients (p values) of protein expression levels in tubular adenomas and hyperplastic polyps. (DOCX)

## Abbreviations

BAX: BCL2-associated X protein
BCL2: B-cell CLL/lymphoma 2
CASP3: activated caspase 3 apoptosis-related cysteine peptidase
CI: confidence interval
CIMP: CpG island methylator phenotype
dMMR: deficient mismatch repair
GIPRF: Gastrointestinal Pathology Research Facility
H&E: Hematoxylin and eosin
HHP: hyperplastic polyp
LI: labeling index
MSI-H: microsatellite instability-high
p16: cyclin-dependent kinase inhibitor 2A
p21: cyclin-dependent kinase inhibitor 1A
SSL: sessile serrated lesion (World Health Organization Classification of Tumours, 5th Edition, 2019)
SSLD: sessile serrated lesion with dysplasia
TMA: Tissue Microarray
TSA: traditional serrated adenoma
UT-MDACC: University of Texas M. D. Anderson Cancer Center
