# Supplementary material for: Proliferation, apoptosis and their regulatory protein expression in colorectal adenomas and serrated lesions
Source: PLoS One. 2021 Nov 11;16(11):e0258878. doi: 10.1371/journal.pone.0258878 (PMC8584700; doi:10.1371/journal.pone.0258878)
Supplement: S1 Table — (DOCX) [file pone.0258878.s001.docx]

**S1 Table:** List of proliferation and apoptosis proteins examined in this study

| **Protein** | **Name** | **Pathway** | **Localization and parameter** |
| --- | --- | --- | --- |
| Ki-67 | Ki-67 | Proliferation index | Nucleus, labeling index % |
| Cyclin D1 | Cyclin D1 | Proliferation (pro) | Nucleus, labeling index % |
| p16 | Cyclin-dependent kinase inhibitor 2A | Proliferation (anti) | Nucleus, labeling index % |
| p21 | Cyclin-dependent kinase inhibitor 1A | Proliferation (anti) | Nucleus, labeling index % |
| CASP3 | Activated caspase 3 apoptosis-related cysteine peptidase | Apoptosis index | Nucleus, labeling index % |
| BAX | BCL2-associated X protein | Apoptosis (pro) | Cytoplasm, labeled area um^2^ |
| BCL2 | B-cell CLL/lymphoma 2 | Apoptosis (anti) | Cytoplasm, labeled area um^2^ |
| Survivin | Survivin | Apoptosis (anti) | Nucleus, labeling index % |
