# Supplementary material for: Proliferation, apoptosis and their regulatory protein expression in colorectal adenomas and serrated lesions
Source: PLoS One. 2021 Nov 11;16(11):e0258878. doi: 10.1371/journal.pone.0258878 (PMC8584700; doi:10.1371/journal.pone.0258878)
Supplement: S2 Table — (DOCX) [file pone.0258878.s002.docx]

**S2 Table:** List of immunohistochemistry reagents and methods

| **Protein** | **Antibody** | **Vendor** | **Antibody type** | **Antigen retrieval** | **Dilution** |
| --- | --- | --- | --- | --- | --- |
| Cyclin D1 | SP4 | Thermo Fisher (Lab Vision), Freemont, CA | Rabbit monoclonal | Tris-EDTA, 35 minutes | 1:40 |
| p16 | E6H4 | Ventana Medical Systems, Tucson, AZ | Mouse monoclonal | Citrate, 10 minutes | 1:3 from prediluted |
| p21 | WAF1 | Leica Microsystems (Novocastra), Buffalo Grove, IL | Mouse monoclonal | Tris-EDTA, 25 minutes | 1:25 |
| Ki-67 | MIB-1 | Dako, Carpinteria, CA | Mouse monoclonal | Tris-EDTA, 20 minutes | 1:100 |
| BCL2 | 100/D5 | Leica Microsystems (Novocastra), Buffalo Grove, IL | Mouse monoclonal | Citrate, 20 minutes | 1:200 |
| BAX | 2D2 | Invitrogen, Camarillo, CA | Mouse monoclonal | Citrate, 10 minutes | 1:200 |
| Survivin | NB500-201 | Novus Biologicals, Centennial, CO | Rabbit polyclonal | Tris-EDTA, 25 minutes | 1:4000 |
| Cleaved Caspase 3 | DC8 | Cell Signaling, Boston, MA | Rabbit polyclonal | Tris-EDTA, 25 minutes | 1:200 |
