# Supplementary material for: Proliferation, apoptosis and their regulatory protein expression in colorectal adenomas and serrated lesions
Source: PLoS One. 2021 Nov 11;16(11):e0258878. doi: 10.1371/journal.pone.0258878 (PMC8584700; doi:10.1371/journal.pone.0258878)
Supplement: S3 Table — (DOCX) [file pone.0258878.s003.docx]

**S3 Table:** Correlation coefficients (p values) of protein expression levels in tubular adenomas and hyperplastic polyps

|  | **Type** | **Caspase-3** | **p16** | **p21** | **Cyclin D1** | **BCL2** | **BAX** | **Survivin** |
| --- | --- | --- | --- | --- | --- | --- | --- | --- |
| **Ki-67** | TA  HPP | 0.26 (<0.0001)  0.23 (0.03) | 0.13 (0.002)  0.21 (0.05) | 0.25 (<0.0001)  0.15 (0.13) | 0.15 (0.0004)  0.50 (<0.0001) | 0.02 (0.51)  0.04 (0.74) | 0.04 (0.34)  0.12 (0.23) | 0.28 (<0.0001)  0.33 (0.0007) |
| **Caspase-3** | TA  HPP |  | 0.21 (<0.0001)  0.11 (0.30) | 0.27 (<0.0001)  0.15 (0.15) | 0.05 (0.28)  -0.06 (0.55) | -0.09 (0.05)  -0.22 (0.04) | 0.002 (0.96)  -0.07 (0.49) | 0.04 (0.35)  0.0005 (1.00) |
| **p16** | TA  HPP |  |  | 0.25 (<0.0001)  0.24 (0.02) | 0.24 (<0.001)  0.21 (0.04) | 0.04 (0.37)  -0.20 (0.06) | 0.11 (0.01)  0.06 (0.54) | 0.11 (0.01)  0.40 (<0.001) |
| **p21** | TA  HPP |  |  |  | 0.33 (<0.0001)  0.38 (0.0001) | -0.17 (<0.0001)  -0.12 (0.23) | 0.12 (0.006)  0.02 (0.81) | 0.24 (<0.0001)  0.39 (<0.0001) |
| **Cyclin D1** | TA  HPP |  |  |  |  | 0.15 (0.0005)  0.14 (0.16) | 0.18 (<0.0001)  0.15 (0.88) | 0.38 (<0.0001)  0.57 (<0.0001) |
| **BCL2** | TA  HPP |  |  |  |  |  | 0.48 (<0.0001)  0.09 (0.35) | 0.29 (<0.0001)  0.02 (0.85) |
| **BAX** | TA  HPP |  |  |  |  |  |  | 0.26 (<0.0001)  -0.03 (0.72) |

TA, tubular adenomas; HHP, hyperplastic polyps. Data used for Circos diagrams of tubular adenomas and hyperplastic polyps with correlation coefficient threshold > 0.24 and p < 0.0018
